# Small Bowel Obstruction Induced by Concurrent Postoperative Intra-Abdominal Adhesions and Small Bowel Fecal Materials in a Young Dog

**DOI:** 10.3390/vetsci8050083

**Published:** 2021-05-12

**Authors:** Jae-Eun Hyun, Hyun-Jung Han

**Affiliations:** 1 Department of Veterinary Internal Medicine, Konkuk Veterinary Medical Teaching Hospital, Konkuk University, Seoul 05029, Korea; jaeeunhyun@konkuk.ac.kr; 2 Department of Veterinary Emergency Medicine, Konkuk Veterinary Medical Teaching Hospital, Konkuk University, Seoul 05029, Korea

**Keywords:** dog, small-bowel obstruction, postoperative intra-abdominal adhesion, small-bowel fecal sign, anastomosis, autologous peritoneal graft

## Abstract

A 7-month-old neutered male poodle dog presented with general deterioration and gastrointestinal symptoms after two separate operations: a jejunotomy for small-intestinal foreign body removal and an exploratory laparotomy for diagnosis and treatment of the gastrointestinal symptoms that occurred 1 month after the first surgery. The dog was diagnosed as having small-bowel obstruction (SBO) due to intra-abdominal adhesions and small-bowel fecal material (SBFM) by using abdominal radiography, ultrasonography, computed tomography, and laparotomy. We removed the obstructive adhesive lesion and SBFM through enterotomies and applied an autologous peritoneal graft to the released jejunum to prevent re-adhesion. After the surgical intervention, the dog recovered quickly and was healthy at 1 year after the surgery without gastrointestinal signs. To our knowledge, this study is the first report of a successful treatment of SBO induced by postoperative intra-abdominal adhesions and SBFM after laparotomies in a dog.

## 1. Introduction

Postoperative intra-abdominal adhesions (PIAs) are inevitable complications of major abdominal surgery in both humans and animals, with various extents of adhesion formation depending on the patient [1,2,3]. Among human patients, up to 93% develop intra-abdominal adhesions postoperatively, and up to 10% who underwent laparotomy presented with adhesions related to complications such as small-bowel obstruction (SBO) [4]. On the other hand, in small animals, PIA rarely causes serious clinical problems because the active fibrinolytic system in dogs and cats usually prevents adhesion formation, which causes clinical problems after laparotomy [1]. However, if the dogs and cats present clinical and imaging signs of gastrointestinal obstruction after laparotomy, PIAs should be also considered as a differential diagnosis of bowel obstruction in spite of the small possibility [1].

With computed tomography (CT), small-bowel fecal material (SBFM) was first described in humans as “small-bowel fecal signs (SBFSs)”; particulate matter was shown to be mixed with gas bubbles in the lumen of dilated small-bowel loops [5]. SBFM was characterized by the accumulation of undigested matter in the small intestine that resembled the fecal content in the large intestine as a result of delayed transit through the intestinal tract [6]. SBFSs are mainly observed in patients with SBO, but can also occur in patients with metabolic or infectious diseases that cause abnormalities in the small intestine [7]. SBO can result from a variety of causes, such as ingestion of foreign materials, infection, intussusception, postoperative adhesions, or neoplasia. Although abdominal radiography and ultrasound still have high diagnostic value, abdominal computed tomography (CT) is a useful tool for confirmation of obstruction, localization of the site and level, and detection of strangulation and the cause of obstruction [8].

This case report describes the successful surgical treatment of and preventive measures against SBO induced by two unusual postoperative complications, PIA and SBFM, in a young dog. To the best of our knowledge, this is the first report to describe the treatment and outcome of concurrent PIA and SBFM occurring after laparotomies in a dog.

## 2. Case History

A 7-month-old neutered male poodle dog with a body weight of 3.0 kg was presented with anorexia, weight loss, and no defecation at the Konkuk Veterinary Medical Teaching Hospital. According to the referring veterinarian, the dog had been diagnosed with a small-intestinal foreign body (earmuff), which was removed via jejunotomy with 3-0 polyglyconate (Maxon^®^, Davis and Geck, Danbury, CT, USA) 2 months before referral. One month after the operation, the constant diarrhea, vomiting, and anorexia had not improved with symptomatic treatment, including fluid therapy, antiemetics, and antibiotics. Imaging examinations, including radiography and abdominal ultrasonography, showed no foreign bodies, but showed decreased gastrointestinal motility. As the owner suspected that the dog might have eaten foreign bodies again, the referring veterinarian performed another exploratory laparotomy and reported jejunal adhesions and dilatation without any foreign bodies. After the second laparotomy, the dog’s condition deteriorated rapidly, with persistent gastrointestinal signs, including anorexia, vomiting, and weight loss from 3.9 to 3 kg. The dog did not defecate from that point on.

**Figure 1:**
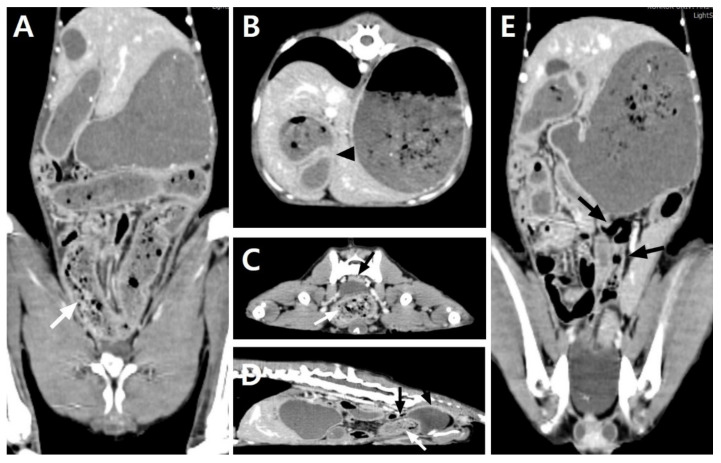
Postcontrast dorsal (**A**,**E**), transverse (**B**,**C**), and sagittal (**D**) computed tomography images of the abdomen of the dog with severe dilatation of the overall small intestines. The stomach and overall jejuna are markedly dilatated, and the proximal jejuna are plicated at an acute angle, causing a stenotic lumen (**B**, black arrowhead). The small-bowel fecal signs show hyper-attenuated fecal-like materials mixed with gas bubbles from the dilated jejunal segments to the entire ileum (**A**,**C**,**D**, white arrows). The large intestines from the ileocecocolic junction to the rectum are collapsed, with little gas and no fecal contents (**C**–**E**, black arrows).

A physical examination on presentation revealed decreased appetite, depressed mentation, mild abdominal pain on palpation, hyperthermia (39.8 °C), and 8% dehydration. The dog’s perfusion parameters were within the normal limits. Hematology revealed normocytic normochromic anemia (packed cell volume (PCV), 28.3%; reference range, 37–55%), leukocytosis (28.83 × 10^9^/L; reference range, 6.0–17.0 × 10^9^/L), and neutrophilia (24.36 × 10^9^/L; reference range, 3.0–11.5 × 10^9^/L), with some toxic changes and reactive lymphocytes. A biochemical analysis, gas analysis, and coagulation test revealed no abnormalities except the elevated C-reactive protein (CRP) level (3.6 mg/dL; reference range, <1.0 mg/dL). Abdominal radiography revealed loss of serosal detail and small-intestinal dilation, with the ratio between the maximal small-intestine (SI) diameter and the height of the fifth lumbar vertebral body (L5) increasing to 2.24. Abdominal ultrasonography revealed a severe dilation with intraluminal intestinal contents and accumulation of fluid in almost the whole jejunal segment, with its largest diameter being 18 mm, as well as jejunal lymph node enlargement. Markedly reduced gastrointestinal motility was generally shown, but remarkable pathological changes were not identified in other gastrointestinal segments except for the jejunum. No foreign material was identified in any segment. A small amount of peritoneal effusion around the urinary bladder and mild hyperechoic change in the mesentery were thought to be due to the previous operation. To identify the cause of the severe jejunal dilatation and the presence and level of the obstacle, a CT scan (LightSpeed; GE Medical System, Milwaukee, WI, USA) was obtained under general anesthesia. The imaging protocols were 120 kVp, 200 mAs, 512 × 512 matrix, and 0.6 rotation time with a 1.25 mm slice thickness. For a contrasting CT examination after the plain CT scan, iohexol (Omnihexol 300; Korea United Pharmaceutical, Seoul, Korea) was manually injected at 600 mg iodine/kg into the cephalic vein. All CT images were transferred to a workstation using a commercially available DICOM imaging analysis software (Osirix viewer; Pixmeo, Los Angeles, CA, USA). The abdominal CT scan revealed generalized gastrointestinal dilatation with abrupt narrowing of the jejunal segment and SBFS (Figure 1). The small intestines were plicated and tortuous, and the jejunal segment was abruptly narrowed to a 4.0 mm diameter. The stomach, duodenum, and jejunal loop proximal to the narrowed lesion were moderately to severely dilated, representing a jejunum with a 21.9 mm maximal diameter. The jejunum distal to the narrowed lesion was normally to mildly dilated, within 8.7 mm in diameter. An SBFS consisting of intraluminal particulate-like materials mixed with gas bubbles was observed within the dilated jejunal segments distal to the narrowed lesion and entire ileum. The lumen of the dilated stomach contained food residues, but there were no remarkable changes in wall thickness or patency. The large intestines, including the cecum, colon, and rectum, were collapsed without fecal contents. A small quantity of free fluid around the dilated jejunal segment and a diffusely hyper-attenuated mesentery were identified. On the basis of the imaging findings, the dog was diagnosed as having SBO of the jejunal segment caused by the stenotic jejunal lumen. The SBO was estimated to be of high grade, with >50% discrepancy between the proximal and distal small-bowel luminal calibers based on the previously reported human classification system [9]. No causes that could induce SBO, such as foreign bodies or other intestinal wall abnormalities, were identified.

**Figure 2:**
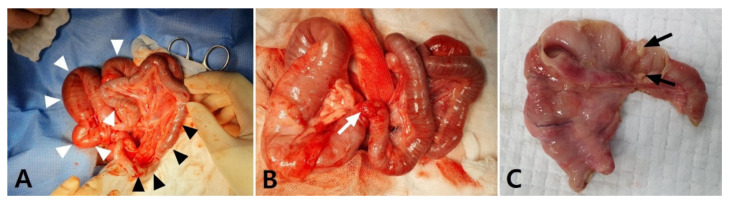
Intraoperative photographs of a dog that presented with postoperative small-bowel obstruction with small-bowel fecal signs in computed tomography images. (**A**) The proximal jejuna were adhered, inducing plication at acute angles (white arrowheads); they were also markedly dilated and hyperemic. The distal jejunum and ileum were mildly dilatated and contained hard intestinal contents that could not be moved or crushed (black arrowheads). (**B**) After the release of the entrapped jejunal segments, a fibrinous adhesive strand was observed to have tightened the middle region of the jejunum, inducing jejunal stenosis (white arrow). A large discrepancy in the intestinal lumen was identified between the proximal and distal jejunum to the stenosed lesion. (**C**) Thick, fibrous adhesive tissue developed between the adjacent serosa and reduced the intestinal lumen (black arrows).

To identify and correct the cause of the SBO, an exploratory laparotomy was performed. The dog was premedicated with cefazolin (30 mg/kg intravenously (IV)), butorphanol (0.2 mg/kg IV), midazolam (0.1 mg/kg IV), and maropitant (1 mg/kg SC). General anesthesia was induced with propofol (4 mg/kg IV) and maintained with isoflurane in oxygen after endotracheal intubation. After an abdominal midline incision was made, diffuse fibrous adhesions were identified throughout the overall jejunum and between the jejunum and peritoneal wall. Bowel plication was identified between the proximal jejunums, adhering to the serosal layers with loops positioned at acute angles, causing the narrowing of the lumen. The jejunal loop proximal to the narrowed lesion was markedly distended and hyperemic, and the distal jejunum and ileum distal to the narrowed lesion were obstructed with hard intestinal contents that could not be squeezed (Figure 2A). The large intestines distal to the obstructed ileum were totally collapsed with no palpable contents. Adhesiolysis was performed using blunt dissection and electrotomy. After the adhesiolysis, a stenosed jejunal lesion was identified, with a fibrinous adhesive strand tightly encircling the narrowed jejunum, inducing a SBO (Figure 2B). The near-complete obstruction of the lesion led to a wide difference between the proximal and distal jejunal calibers with lack of luminal patency. The stenosed jejunum was removed and anastomosed using a previously described sutured anastomosis [10]. The resected intestinal segment was grossly inspected. The antimesenteric border was carefully incised to observe the adhesive stenosed lesion that caused the SBO, and a strong, thick, fibrous tissue that formed between the adjacent intestinal serosa was identified to markedly reduce the jejunal lumen (Figure 2C). No other obvious gross abnormalities were observed in the affected jejunum. By direct observation and palpation, it was confirmed that there were no pathological changes or impaired patency of the stomach or other intestinal segments. Cytology and bacterial culture using a small amount of abdominal effusion and adhesive peritoneum revealed no bacterial growth, but a histopathological analysis of the resected intestine was not performed because it was a financial burden to the owner.

**Figure 3:**
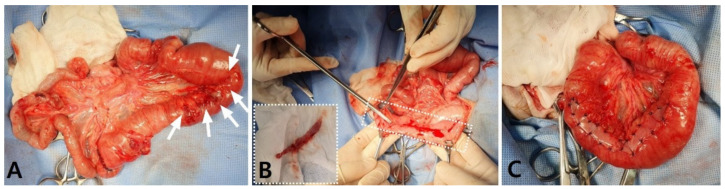
Intraoperative photographs representing the damaged serosal surface of the jejunum after adhesiolysis in a dog. (**A**) The proximal jejunum, which was released after adhesiolysis, represents the damaged serosal surface of approximately 7 cm in length (white arrows). (**B**) A parietal peritoneum (8 × 1.5 cm rectangular shape) was harvested from the left side of the abdominal wall. (**C**) The excised peritoneal graft was attached to the injured serosal surface with simple interrupted sutures to cover the entire damaged serosa.

The hardened fecal-like contents were removed via the enterotomies that were made over the distal end of the jejunum and ileum and were identified as hard and dry fecal matter corresponding to a fecal score of 1. The enteronotomy sites were closed with 3-0 polydioxanone (PDS) in a simple interrupted pattern. After securing the intestinal patency and confirming that there was no leakage, the serosal surfaces of the entire small intestine were closely examined, and a remarkable serosal injury of 7 cm in length was identified in the proximal jejunum where the adhesion was separated (Figure 3A). To prevent re-adhesion of the damaged serosa, an autologous peritoneal graft (APG) was transplanted to cover the entire damaged lesion. As an APG, 8 × 1.5 cm of the parietal peritoneum was harvested at the middle of the left-side of the abdomen (Figure 3B). The APG was expanded over the injured serosal surface and attached with several simple interrupted sutures using 3-0 PDS II (Ethicon, Somerville, NJ, USA; Figure 3C). The margin of the excised parietal peritoneum was apposed by a simple continuous suture using 4-0 PDS II (Ethicon). The abdomen was fully lavaged with warm saline and closed routinely.

Postoperatively, the dog recovered from anesthesia uneventfully. He was quiet, alert, and responsive, and he maintained normal vital signs and normotension. His postoperative medication included continuous infusion of fentanyl (4 μg/kg/h) and lidocaine (50 μg/kg/h) for the first 24 h, followed by carprofen (2.2 mg/kg orally twice daily) and tramadol (5 mg/kg orally twice daily) for 5 days, maropitant (0.1 mL/kg subcutaneous once daily) for 4 days, and misoprostol (5 μg/kg orally twice daily) for 5 days. As intestinal supplements, probiotics were administered for 2 weeks after the operation. During hospitalization after the surgery, the dog showed no vomiting, regained his appetite, started to defecate (fecal score: 5/7) from postoperative day 2, and was discharged on postoperative day 4.

Postoperative follow-up examinations, including radiography, ultrasonography, and blood examination, showed no dilatation, obstruction, or plication, with normal motility of the overall intestines. Regenerative anemia, mild hypoalbuminemia, neutrophilic leukocytosis, and elevated CRP levels were identified immediately after surgery, but returned to within the reference ranges in 2 weeks. Throughout the one-year telephone follow-up, the dog remained healthy without any clinical signs associated with gastrointestinal disease. Considering the history of two separate previous operations, the confirmation that there were no other obvious pathological changes, including foreign bodies, and the fact that there was no recurrence after resecting the obstructive jejunum, this patient was tentatively diagnosed with SBO induced by PIA.

## 3. Discussion

PIA occurs when the balance between fibrin deposition and fibrinolysis, which proceeds normally in the case of peritoneal injury, is disrupted by specific causes. The peritoneum has powerful coagulation and fibrinolytic capacity, which normally exceeds coagulation; thus, abdominal adhesion does not commonly occur under normal conditions [11]. As the integrity of the mesothelial layer constituting the peritoneum promotes fibrinolytic activity, mesothelial damage is considered to accelerate peritoneal adhesion formation by reducing the fibrinolytic capacity to lower than the coagulation function of the peritoneum [12,13].

In human medicine, PIAs are the most frequent causes of SBO, of which adhesions account for 50–75% of SBOs, which occur after surgery in 80% of all cases [8]. Early postoperative SBOs caused by PIAs occur within 30 days after surgery [14,15]. This is a familiar but challenging complication for surgeons, with an incidence of approximately 1–12% in human patients undergoing abdominal surgery [14,15]. As PIA-induced SBO can be difficult to distinguish from functional ileus, which typically occurs after abdominal surgery, CT could be beneficially used to confirm the adhesive obstruction while checking for other causes of mechanical obstruction and localizing the site and level of the obstruction [8,16,17]. The widespread use of CT and awareness of the high incidence of PIA-induced SBO can help surgeons recognize early postoperative SBOs and establish appropriate treatment strategies, especially if the apparent causes of mechanical SBOs could not be identified in patients with a history of prior surgery [16]. Compared with several human studies [18,19,20], studies in veterinary medicine have paid little attention to PIAs and associated SBOs, and studies of PIA-induced SBOs in small animals are lacking. In the present case, a PIA-induced SBO was highly suspected due to the dog’s history and CT scans, and it was confirmed on the basis of the gross morphology during the surgery. The dog presented with persistent gastrointestinal signs and markedly reduced gastrointestinal motility on ultrasonography after the laparotomies, so a typical postoperative functional ileus was initially considered. However, marked dilatation of the small bowel without obvious causes of an SBO, such as foreign bodies or tumors, was identified, and the CT revealed similar images of PIA-induced SBOs in human studies, including mechanical SBOs with no obvious causes, proximal dilated loops of the small bowel, and an undilated distal bowel with a transition zone [8]. With this history and CT findings, the typically occurring postoperative functional ileus and other causes of mechanical SBO could be excluded. PIA, which was suspected to have resulted from the two inexperienced open abdominal surgeries, was considered as the main cause of the SBO in this dog.

Human patients with PIA-induced SBOs usually improve with non-surgical treatment, and 80–100% of patients experience alleviation of their clinical signs within 2 weeks of the symptoms’ onset after non-surgical treatment [15,21]. On the other hand, a significant number of patients with PIA-induced SBOs (14%–58%) still require surgical treatment because of clinical and radiological symptoms that indicate intestinal strangulation or obstruction [15,16]. In addition, the duration of non-surgical treatment and nature of the index operation also affect the decision for surgical treatment [16]. As fibroblast contents increase 2 weeks after surgical injury, thus inducing the maturation of adhesion [16,21], surgical treatment may become more invasive after 2 weeks of the injury. In a previous human study, patients who underwent late surgery (>13 days after surgical peritoneal injury) had a higher enterotomy rate of 17%, whereas those who underwent surgery before 13 days presented a 5% rate [22]. On the basis of these human studies, in small animals with suspected PIA-induced SBOs, surgical treatment may be fully considered as a valid option if symptoms do not improve within 2 weeks in spite of non-surgical management. Moreover, an operator should be mindful that the surgery may become hazardous as time passes, especially if it is to be performed after more than 2 weeks after the previous surgery. In this dog, a relatively long time—over 1 month—had passed before surgery was performed for the management of the PIA-induced SBO. Consequently, the severe adhesive lesions that induced the irreversible jejunal stenosis were identified; thus, jejunal resection and anastomosis had to be performed to regain the intestinal patency. If the dog had been surgically treated a little earlier, before the adhesion maturation had progressed further, the invasiveness of the surgery could have been reduced. Unfortunately, the association between PIA and SBO in canine patients has not been reported as much as that in humans. Therefore, further studies are needed on the prevalence and pathological period of PIA-induced SBOs in dogs.

Furthermore, SBFSs were identified in the CT results of the dog, and hardened fecal materials in the distal jejunum and ileum were confirmed during surgery. This was first described in human beings in 1995 [5], but no study has reported SBFSs in the field of veterinary medicine. SBFSs are mainly due to the SBO inducing stagnation of the intestinal contents, which causes gradual fluid absorption across the bowel wall, leaving undigested fecal matter to accumulate in the SI [6]. In human patients with SBOs due to adhesions, the prevalence of SBFSs ranged from 37.1% to 55.9% [9,23,24,25], despite the earliest study reporting an SBFS prevalence of 7.4% among SBO cases [5]. Due to the high prevalence, routine CT examination is recommended to identify SBFSs in human patients with SBOs [23]. In this dog, the SBFS was identified in the distal jejunum and ileum distal to the stenotic lesion in CT images and surgery. In this dog, the SBFS was located distal to the transition zone, which indicated a region undergoing a radical change in intestinal luminal diameter due to the obstructed lesion, whereas it is usually (93%) identified immediately proximal to the transition zone [23]. The reason for this difference was estimated to be the reduced overall intestinal motility and decreased amount of water in the intestinal contents distal to the stenotic lesion. In this dog, the distal jejunum and ileum were not expected to have severe intestinal stasis due to the mechanical obstruction because they were distal to the stenotic lesion; however, postoperative functional ileus might have more likely induced the SBFM in this dog in that markedly reduced intestinal motility could lead to overall intestinal stasis, resulting in increased fluid absorption across the bowel wall. In addition, as the intestines distal to the stenosis were not severely distended, the amount of water in the intestinal contents was not as much as that in the severely distended intestine proximal to the stenosis; thus, the drying and hardening of fecal matter might have occurred relatively quickly. The degree of SBO in the dog of this study was assessed using a human grading system based on the discrepancy between the proximal and distal small-bowel luminal calibers. Further research is needed to establish an imaging occlusion grading system for diagnosis and prognosis of canine SBO patients [9].

The clinical significance of SBFSs in human medicine remains unclear. Although the relevance of correlation between SBFSs and diagnosis of SBO remains controversial in patients with acute abdominal pain, it has been reported that SBFSs are useful signs for localization of transition zones in SBO patients on account of their tendency to be most prominent in the transition region from the dilated intestinal loop to the collapsed bowel [9]. SBFSs do not independently support the prediction of successful treatment or progression of lesions such as ischemia [23]. However, as in this dog, hardened fecal matter in the SI could cause mechanical obstruction and additional intestinal damage, such as ischemia, which would require surgery. Therefore, surgical removal of the SBFM may be necessary for the management of SBO in dogs. A model for criteria to predict the need for operative intervention was presented in human medicine, and was also demonstrated to be proportional to the morbidity and mortality [26]. In this model, patients with mesenteric edema, lack of an SBFS, and obstipation were strongly recommended for early operative exploration. Further studies are needed to establish criteria for the presence and timing of surgery in canine SBO patients.

PIA-induced SBO has been considered as a high-risk recurring problem after surgical correction in human studies (B), which reported overall recurrence rates ranging from 8.7% to 53% [19,27,28,29,30], while related studies in small animals are lacking. The previously reported risk factors for increasing re-adhesion included the magnitude of the surgical trauma in both the parietal peritoneum and intestinal serosa, decreased intraperitoneal fibrinolytic activity, and early patient age [11,31,32,33]. Owing to this high prevalence of recurrence, extensive research for preventing re-adhesion has been conducted, but no clinical standard has been established for any preventive modalities, either surgical or pharmacological [13,34]. As one of these preventive modalities, barriers, which are a kind of a membrane or gel developed to separate the damaged serosal surfaces from the adjacent organs [3,20], have been demonstrated to be effective in animal models and clinical trials [3,20,34]. Most recently, APG was demonstrated to have a preventive effect on peritoneal adhesion by transplanting mesothelial cells and rapid reperitonealization [34]. Mesothelial cells form the thin basement membrane of the peritoneum and are supported by a submesothelial layer consisting of a connective tissue stroma [35]. The mesothelial cells maintain the serosal integrity, and their progenitor cells could play an important role in peritoneal remodeling [35]. Thus, preservation of functional mesothelial cells is a necessary factor for adhesion prevention, and an APG can provide mesothelial reconstruction and preserve the function of the transplanted mesothelial cells [34]. In addition, this autologous barrier has other benefits, including minimizing adverse biological reactions, availability, and economic feasibility, as compared with synthetic barriers [34]. In this dog, a meticulous surgical technique, including careful tissue handling to avoid desiccation and ischemia, was established as the first step to prevent re-adhesion. Moreover, the significant serosal damage in the released jejunum was covered with an APG to separate the damaged serosa from the other adjacent intestines to prevent re-adhesion. On the basis of the study by Bresson et al. [34], the mesothelial layer of the graft should be exposed to the abdominal cavity during transplantation because the appropriately positioned polarity of the mesothelial cells is important for preserving functional mesothelial cells [33]. They reported that the mesothelial cells were detected 14 days after APGs were transplanted with the mesothelial cell side exposed to the abdominal cavity, but were not detected when the submesothelial layer was exposed to the abdominal cavity [34]. Although the preventive effect of APGs on serosal re-adhesion has been proven in human patients and in rat and mouse models [3,20,34], an additional randomized, prospective study is necessary in order to establish the evidence of the prophylactical application of APGs in canine patients.

## 4. Conclusions

This study describes the first reported case of PIA and obstructive SBFM-induced SBO in a young dog. These two rarely occurring postoperative complications were diagnosed on the basis of the dog’s history and CT results and were successfully treated with meticulous surgical techniques for resection of the obstructive adhesive lesion and removal of the obstructive SBFM via enterotomies. The deperitonealized lesion of the released jejunum was completely covered with autologous peritoneal graft to prevent re-adhesion.

## Data Availability

Data sharing is not applicable.
